# CXCL1-Triggered Interaction of LFA1 and ICAM1 Control Glucose-Induced Leukocyte Recruitment during Inflammation *In Vivo*

**DOI:** 10.1155/2012/739176

**Published:** 2012-10-09

**Authors:** Kirsten Buschmann, Lutz Koch, Natascha Braach, Hanna Mueller, David Frommhold, Johannes Poeschl, Peter Ruef

**Affiliations:** Clinic of Neonatology, Department of Pediatrics, University of Heidelberg, 69120 Heidelberg, Germany

## Abstract

It is well acknowledged that proinflammatory stimulation during acute hyperglycemia is able to aggravate inflammatory diseases. However, the mechanisms of proinflammatory effects of glucose are controversially discussed. We investigated leukocyte recruitment after intravenous injection of glucose in different inflammatory models using intravital microscopy. Flow chamber experiments, expression analysis, functional depletion, and knockout of key adhesion molecules gave mechanistic insight in involved pathways. We demonstrated that a single injection of glucose rapidly increased blood glucose levels in a dose-dependent manner. Notably, during tumor necrosis factor (TNF) **α**-induced inflammation leukocyte recruitment was not further enhanced by glucose administration, whereas glucose injection profoundly augmented leukocyte adhesion and transmigration into inflamed tissue in the trauma model, indicating that proinflammatory properties of glucose are stimulus dependent. Experiments with functional or genetic inhibition of the chemokine receptor CXCR2, intercellular adhesion molecule 1 (ICAM1), and lymphocyte function antigen 1 (LFA1) suggest that keratino-derived-chemokine CXCL1-triggered interactions of ICAM1 and LFA1 are crucially involved in the trauma model of inflammation. The lacking effect of glucose on **β**_2_ integrin expression and on leukocyte adhesion in dynamic flow chamber experiments argues against leukocyte-driven underlying mechanisms and favours an endothelial pathway since endothelial ICAM1 expression was significantly upregulated in response to glucose.

## 1. Introduction

Although proinflammatory effects of acute hyperglycemia in inflammatory conditions and septic patients have been extensively investigated in clinical as well as experimental settings, there are still controversies about its relevance and mechanisms [1, 2]. In contrast to earlier studies Brunkhorst et al. found no beneficial effect of treating acute hyperglycemia in the clinical setting of sepsis [3]. However, in this context it was difficult to dissect between effects directly attributable to insulin or thereby induced hypoglycemia. Similarly, hyperglycemia-related proinflammatory effects should be separated into those directly evoked by glucose and those induced by secondary hyperglycemia (stress, inflammation). In case of acute hyperglycemia caused by critical illness, altered secretion of counterregulatory hormones and excessive release of proinflammatory cytokines is observed as well as suppression of the innate immune system [4, 5]. These consequences of impaired leukocyte function are in part comparable to the pathophysiology observed in patients with diabetes mellitus. Despite tremendous research on proinflammatory conditions related to hyperglycemia direct effects of glucose on leukocyte recruitment during inflammation are poorly studied [6–8].

Leukocyte recruitment into inflamed tissue follows a well-defined cascade of events beginning with the capture of free flowing leukocytes to the vessel wall followed by leukocyte rolling along and adhesion to the inflamed endothelial layer [9, 10]. During rolling, leukocytes get into intimate contact with the endothelial surface, which allows endothelial bound chemokines (i.e., CXCL1) to interact with their specific chemokine receptors (i.e., CXCR2 as the main chemokine receptor on neutrophil leukocytes) on the leukocyte surface. This triggers the activation of *β*_2_-integrins (i.e., LFA1 and MAC1) which leads to firm leukocyte arrest on the endothelium. In addition, integrin-dependent signaling events induce cytoskeletal rearrangements and cell polarization, modifications necessary in helping to prepare the attached leukocyte to spread and crawl in search for its wayout of the vasculature into tissue [10–13]. After crawling along the vessel wall, extravasation takes place as the last distinctive step of the leukocyte recruitment cascade [14].

Little is known about how glucose might interfere with the cascade of leukocyte recruitment *in vivo* [15]. Acute hyperglycemia severely alters the balance of ana- and catabolic metabolism towards catabolism. Insulin secretion promotes neutrophil chemotaxis under physiological conditions; acute hyperglycemia leads to insulin resistance in and reduced respiratory burst of neutrophil leukocytes [1].

Insulin resistance is seen in different tissues including muscle cells and the substrate producing organ liver. Hepatic insulin resistance leads to an exaggerated release and an increased blood concentration of not only glucose, but also of amino acids and of free fatty acids. Paradoxically, for example, fatty acids are metabolized to a smaller extent in patients with critical illness, leading to a progressively increasing vicious circle of not extractable circulating metabolic substrates [1].

Although experimental design (route and doses of glucose, observed tissue) varied between existing studies, they revealed increased rolling, adhesion and transmigration of leukocytes after glucose application, an effect which was at least in part reversible after insulin injection [7]. Since changes of osmolarity were repeatedly discussed to be causative of alteration of leukocyte recruitment, Azcutia et al. carried out experiments not only with the biological active D-glucose (that is referred to as “glucose” in all existing experimental settings as well as in our investigations), but also with its synthetically existing enantiomer L-glucose [8]. Osmotic changes were not causative in this setting, as L-glucose failed to alter leukocyte recruitment.

Since there are no intravital microscopic studies investigating the immediate effects of glucose on leukocyte recruitment *in vivo*, we aimed to observe leukocyte recruitment in response to intravenous glucose application in different inflammatory mouse models by intravital microscopy. Based on our experimental results we found that in contrast to TNF*α* stimulation leukocyte adhesion and transmigration can be additionally stimulated by glucose, which seems to be dependent on CXCL1-triggered interaction of ICAM1 and LFA1 during trauma-induced inflammation.

## 2. Materials and Methods

ICAM1^−/−^ mice were generated as described earlier and backcrossed for at least seven generations into the C57bl/6 background [16]. ICAM1^−/−^ mice were generated by cross-breeding ICAM1^−/−^ C57bl/6 mice. LFA1^−/−^ and C57bl/6 wildtype (WT) mice were provided by Charles River (Sulzfeld, Germany). All mice were maintained as breeding colonies at the Central Animal Facility of the University of Heidelberg, Germany. For intravital microscopy experiments, mice were at least 8 weeks of age. The animal experiments were approved by the Animal Care and Use Committee of the Regierungspräsidium Karlsruhe, Germany.

In certain experiments, recombinant murine TNF*α* (R&D systems, Minneapolis, USA) was injected intrascrotally at a dose of 500 ng per mouse 3 hours before intravital microscopy. In some experiments, recombinant murine chemokine CXCL1 (keratinocyte-derived chemokine KC; Peprotech, London, UK) was injected systemically at a dose of 600 ng per mouse. Blocking antibodies against murine MAC1 (Tib128, clone M1/70, rat IgG2b) and murine LFA1 (Tib217, clone M17/4, rat IgG2a) were obtained from American Type Culture Collection (ATCC, Manasses, USA) and systemically administered with a dose of 100 *μ*g per mouse. *Bordetella pertussis* PTx was purchased from Sigma-Aldrich (Taufkirchen, Germany) and administered in certain experiments at a dose of 4 *μ*g per mouse 3 h prior to preparation in the trauma model.

### 2.1. Intravital Microscopy

Mice were prepared for intravital microscopy as reported recently [17]. Briefly, after intraperitoneal (i.p.) injection of ketamine (125 mg/kg body weight, Ketalar; Parke-Davis, Morris Plains, USA) and xylazine (12.5 mg/kg body weight; Phoenix Scientific, Inc., St. Joseph, USA) mice were placed on a heating pad to maintain body temperature. Intravital microscopy was conducted on an upright microscope (Leica; Wetzlar, Germany) with a saline immersion objective (SW40/0.75 numerical aperture, Zeiss, Jena, Germany). Mice were intubated, and the left carotid artery was cannulated for blood sampling and the right jugular vein for glucose and systemic mAb administration. D-glucose (200 mg/mL) was administered in doses of 0,25 g, 0,5 g, and 1 g/kg body weight. The injection of the equivalent volume of normal saline (NaCl 0,9%) served as control agent. Additional osmotic controls were performed with the biologically inactive L-glucose, purchased from Sigma-Aldrich (Taufkirchen, Germany). The labeling “glucose” refers to D-glucose that was used in all other experiments that were carried out. This labeling is applied to the entire manuscript. Blood glucose measurements were carried out throughout the experiment using AccuChek (Roche Diagnostics, Mannheim, Germany) and additionally verified by comparing the results in sodium fluoride samples in the Central Laboratory of the University Hospital Heidelberg (Analysezentrum, Heidelberg, Germany).

### 2.2. Cremaster Muscle Preparation

The surgical preparation of the cremaster muscle was conducted as described previously [17]. Briefly, the scrotum was opened and the cremaster muscle exteriorized. After longitudinal incision and spreading of the muscle over a cover glass, the epididymis and testis were mobilized and pinned aside leading to full microscopic access to the cremaster muscle microcirculation. Cremaster muscle venules were recorded via CCD camera (CF8/1, Kappa, Gleichen, Germany) on a Panasonic S-VHS recorder. The cremaster muscle was superfused with thermocontrolled (35°C) bicarbonate-buffered saline. Postcapillary venules under observation were recorded before and during glucose administration and ranged from 20 to 40 *μ*m in diameter. Systemic blood samples (10 *μ*L) were taken and assessed for white blood cell count before and after experiment. Systemic blood samples (10 *μ*L) were taken after each mAb injection and stained with Türks solution 1 : 10 (Merck, Darmstadt, Germany). In some experiments, recombinant murine chemokine KC (keratinocyte-derived chemokine; Peprotech, London, UK) was injected systemically at a dose of 600 ng per mouse. Systemic leukocyte concentration was analyzed using a hematocytometer and expressed as number of leukocytes per *μ*L of whole blood. Microvascular parameters (venular diameter, venular vessel segment length, and leukocyte rolling velocity) were measured using an image-processing system [18]. Venular centerline red blood cell velocity was measured during the experiment via a dual photodiode and a digital online cross-correlation program (Circusoft Instrumentation, Hockessin, USA). An empirical factor of 0.625 was used to convert centerline velocities to mean blood flow velocities [19]. Wall shear rates (*γ*_*w*_) were estimated as 4.9 (8*v*_*b*_/*d*), where *v*_*b*_ is mean blood flow velocity and *d* the diameter of the vessel [20, 21]. 

### 2.3. Whole Mount Histology

To differentially count intravascular and extravascular leukocytes, cremaster muscle-whole mounts were prepared as described before [22, 23]. Briefly, while the cremaster muscle was still mounted on the stage for intravital microscopy, the tissue was fixed with 4% paraformaldehyde in 0.1 M phosphate buffer (pH 7.4). The cremaster muscle was removed and mounted flat on a superfrost glass slide (Menzel, Braunschweig, Germany), air dried for 5–10 min, and fixed in 4% paraformaldehyde in 0.1 M phosphate buffer (pH 7.4) for 24 h at 4°C. After fixation, the tissue was washed three times in 0.1 M phosphate buffer with 5% ethanol, stained with Giemsa (Sigma) at room temperature for 5 min, and differentiated in 0.01% acetic acid for contrast. The differentiated slides were washed in water, 75% ethanol, 95% ethanol, 100% ethanol, and fresh xylene, followed by mounting in mounting media (AGAR Scientific). The Giemsa-stained cremaster muscles were observed using a Leica DMRB upright microscope and a 25/0,75 NA oil immersion objective (both Leica, Germany). Intravascular and interstitial leukocytes were counted and differentiated into neutrophils, eosinophils, and mononuclear cells.

### 2.4. Immunohistochemistry

To investigate the endothelial expression of ICAM1 on unstimulated cremaster muscle venules or during trauma-induced inflammation, we performed immunohistochemical analysis of whole-mount cremaster muscles as described [24, 25]. Briefly, after insertion of a catheter into the carotid artery, primary antibodies against ICAM1 (YN-1, monoclonal rat anti-mouse; 30 *μ*g/mouse, ATCC, Wesel, Germany) were systemically injected and incubated for 10 minutes. Because of the intravascular antibody application, which was performed before surgical preparation of the cremaster muscle, binding of antibodies is mostly restricted to surface expressed antigens within the vasculature. For unstimulated venules, excess antibody was washed out from the circulation with normal saline solution before the cremaster muscle was obtained. For trauma-stimulated venules, washing out of excess antibody occurred after exteriorization of the cremaster muscle and 20 minutes of superfusion with superfusion buffer. Cremaster muscle whole mounts were surgically prepared as reported previously [24] and transferred onto adhesive slides (Superfrost, Menzel, Braunschweig, Germany). After air-drying over 5–10 min, cremaster muscles were fixed in acetone (−18°C) overnight. Permeabilization of the tissue was conducted in 0.05 mol/L Tris buffer containing 0.003% saponine. After washing, cremaster muscle tissue was incubated with biotin-conjugated secondary goat anti-rat antibody (eBioscience, USA) over 45 min in a humid chamber. Blocking of endogenous peroxidase activity was performed with H_2_O_2_ in methanol over 1 h, followed by incubation of the tissue with peroxidase-conjugated streptavidin. Staining of tissue samples was performed using a commercially available kit (DAB, Vector Lab, Burlingame, USA). For counter-staining Mayer's hemalaun (Merck, Germany) was applied. Thereafter, the samples were incubated with an increasing concentration of alcohol followed by xylene (Carl Roth, Karlsruhe, Germany). Sealing of tissue samples was performed with mounting medium (DPX Mounting Medium, AGAR Scientific, Stansted Essex, UK). 

Analysis of stained slides was conducted on a Leica DMRB upright microscope and a ×25/0.75 NA oil immersion objective (both Leica, Wetzlar, Germany). Photographs of the samples were taken using a color CCD camera (KAPPA, Germany).

### 2.5. Isolation of Bone Marrow Neutrophils

Murine bone marrow PMNs were isolated from femurs and tibias, as described previously [26]. After isolation, they were loaded on top of a discontinuous Percoll gradient (52%/64%/72%) and centrifuged at 1000 g for 30 minutes. PMNs were harvested from the 64%/72% interface, washed in PBS, and cultivated for 24 hours in RPMI 1640 medium supplemented with 20% WEHI-3B-conditioned medium. PMN viability was greater than 95% as assessed by the trypan blue exclusion test, and purity was greater than 98% as analyzed by microscopy using Hemacolor staining (Merck, Darmstadt, Germany).

### 2.6. Flow Cytometry

The expression of MAC1 and LFA1 on bone marrow-derived neutrophils was assessed by flow cytometry. After red blood cell lysis, 10^6^ leukocytes/mL were stimulated for 15 min with 10 mg glucose at 37°C. Next, cells were incubated in the dark with FITC-conjugated anti-MAC1 mAb M1/70 (1 *μ*g/10^5^ cells, rat IgG2b; eBioscience, San Diego, USA), FITC-conjugated anti-LFA1 mAb M17/4 (1 *μ*g/10^5^ cells, rat IgG2a; eBioscience, San Diego, USA), or respective FITC-conjugated isotype control antibodies (1 *μ*g/10^5^ cells, rat IgG2b or rat IgG2a; eBioscience, San Diego, USA) to detect anti-MAC1 and anti-LFA1 signals, respectively. MAC1 and LFA1 expression was assessed on 10.000 cells per mouse within the neutrophil cluster defined by forward-side scatter analysis using LSRII with DIVA software package (Becton Dickinson, San Jose, USA). Expression of MAC1 and LFA1 upon stimulation with different glucose concentrations was compared to unstimulated cells and their respective isotype controls.

### 2.7. Flow Chamber Assay

Flow chamber experiments were conducted as described [23, 27]. In brief, rectangular microglass capillaries (VitroCom, Mountain Lakes, USA) were coated with rmP-selectin (2 *μ*g/mL), rmCXCL1 (5 *μ*g/mL), and ICAM1 (1 *μ*g/mL) and connected via PE tubing to a 2 mL syringe containing freshly isolated bone marrow neutrophils from *LysEGFP *mice. In *LysEGFP *mice the enhanced GFP (EGFP) is knocked into the murine lysozyme M (*lys*) locus leading to the expression of EGFP in myelomonocytic cells. The cell suspension (0.25 × 10^6^ GFP positive cells) was perfused through the flow chamber and adhesion of GFP-positive cells observed by fluorescence microscopy (BX51 WI with a saline immersion objective × 20/0.95 NA, Olympus Hamburg) for 10 minutes under constant flow conditions using a high precision perfusion pump (Harvard Instruments, March-Hugstetten, Germany; wall shear stress 0.1 Pa). Images were recorded via a CCD camera system (CF8HS; Kappa) on a Panasonic S-VHS recorder. In some experiments, cell suspensions were incubated with D-glucose (10 mg/10^6^ cells) 15 minutes before the perfusion through the flow chamber.

### 2.8. Statistics

Sigma Stat 3.5 (Systat Software, Erkrath, Germany) was used for statistical analysis. Leukocyte counts, vessel diameters, leukocyte adhesion, leukocyte-rolling flux fractions, wall shear rates, and *in vitro* leukocyte adhesion between groups and treatments were compared with one-way ANOVA followed by a multiple pairwise comparison test (Dunn's test) or by Wilcoxon rank-sum test, as appropriate. Statistical significance was set at *P* < 0.05.

## 3. Results and Discussion

### 3.1. Glucose-Dependent Leukocyte Adhesion and Transmigration in Trauma-Induced Inflammation

The first objective of our experiments was triggering a significant increase of the murine blood glucose concentration by intravenous glucose application. As described earlier, stress (induced by anesthesia and surgical preparation) resulted in high basal blood glucose levels in all investigated mice [28]. As expected one minute after glucose injection blood glucose concentration increased significantly in a dose-dependent manner (See Supplemental Table 1 available online at doi:10.1155/2012/739176). In contrast, recent studies [7, 8] did not detect elevation of systemic blood glucose levels presumably due to different experimental setup (intraperitoneal injection of glucose and longer-time interval until microscopic observation). Because we observed a rapid and significant increase of systemic blood glucose concentration after a dose of 0,5 g/kg glucose, which is in the range clinically used to treat severe hypoglycemia [29], we continued with that dose in all further experiments.

Surgical preparation of the cremaster muscle induces leukocyte adhesion mainly via the chemokine CXCL1-CXCR2 pathway and *β*_2_ integrins LFA1 and MAC1 in the short-term model of trauma-induced inflammation [23, 27]. Using this model we analyzed the number of adherent leukocytes in WT mice in postcapillary venules of the cremaster muscle before and after intravenous injection of different doses of glucose. The results obtained from these experiments were compared to injection of normal saline and L-glucose. There were no differences in hemodynamic and microvascular parameters (vascular diameter, blood flow velocity, wall shear rate, and white blood cell count) before and after glucose or saline injection (Table 1). Thereby, we ruled out that alterations of leukocyte recruitment might be caused by hemodynamic changes in response to fluid injection.

**Figure 1:**
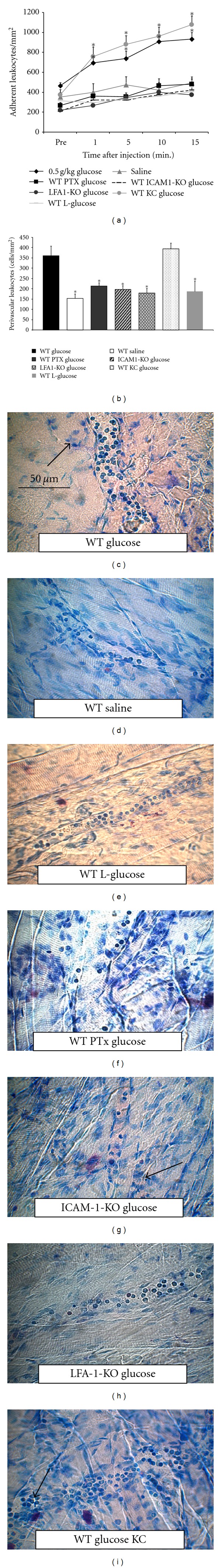
Leukocyte adhesion (number of adherent cells/mm^2^) in mouse cremaster muscle venules and leukocyte transmigration (perivascular neutrophils/mm^2^ surface area) in cremaster muscle whole mounts in response to glucose during trauma-induced inflammation. (a) Leukocyte adhesion was observed in trauma-stimulated cremaster muscle venules of wild-type mice (64 venules in 16 mice), *LFA1-KO *mice (19–25 venules in 4 mice), and *ICAM1-KO* mice (18–22 venules in 4 mice) before and during 15 minutes after intravenous injection of 0.5 g/kg glucose. To dissect the role of the chemokine pathway in this model, WT mice were either pretreated with 4 *μ*g PTx 3 h prior to cremaster exteriorization and glucose stimulation or coinjected with 600 ng CXCL1 (KC) and glucose and compared to injection of normal saline. WT mice injected with the equivalent volume of normal saline (18 venules in 4 mice) and the biologically inactive L-glucose (13–28 venules in 5 mice) served as controls. (b) Giemsa-stained cremaster muscle whole mounts of WT mice were analyzed for the number of perivascular neutrophils (per mm^2^ surface area) after injection of glucose or normal saline in the trauma model). In parallel, leukocyte extravasation was quantified in glucose-treated LFA1-KO, ICAM1-KO mice, and WT mice pretreated with 4 *μ*g PTx 3 h prior to cremaster muscle exteriorization or coinjected with 600 ng CXCL1. To further illustrate the results representative micrographs of cremaster muscle whole mounts are shown in (c)–(i). Reference bar is shown in (c). Arrows point to extravasated leukocytes. All values are presented as mean ± SEM. Significances are indicated by the asterisks (*P < 0.05 versus WT control mice).

Administration of a low dose of 0,25 g/kg glucose did not lead to any significant changes of leukocyte adhesion. However, increasing doses of glucose (0,5 g/kg and 1 g/kg, resp.) lead to a significant, dose-dependent increase of leukocyte adhesion in WT mice within 15 minutes after glucose administration (Figure 1(a)) compared to controls injected with normal saline or L-glucose. These results suggest that glucose triggers leukocyte adhesion which is not attributable to hemodynamic changes.

To investigate the impact of glucose on neutrophil transmigration, we also performed Giemsa staining of whole-mount cremaster muscles in this model and classified leukocytes into neutrophils, eosinophils, and mononuclear cells as described [17]. Accordingly, the number of perivascular neutrophils was higher after administration of glucose compared to saline controls suggesting that glucose-triggered leukocyte adhesion translates into increased transmigration of leukocytes, too (Figures 1(b)–1(d)). Taken together, leukocyte recruitment during trauma induced inflammation can be rapidly enhanced by intravenous glucose injection. Despite different experimental design, this observation is consistent with intravital microscopic studies on mesenterial vessels of Schäffler et al., Booth et al., and Azcutia et al. [6–8]. Importantly, neither changes of hemodynamic conditions nor osmolarity [6, 8] are causative for the observed glucose induced effects. Moreover, there is evidence that glucose triggered leukocyte recruitment is reversible through insulin treatment [7].

### 3.2. Glucose-Dependent Leukocyte Adhesion and Transmigration during TNF*α*-Induced Inflammation

As a potent proinflammatory agent, we administered *TNF*α** in a dose of 500 ng 3 h prior to exteriorization of the cremaster muscle and observed leukocyte adhesion in cremaster muscle venules of WT mice, either treated with glucose or normal saline. In this model, transition from leukocyte rolling to firm adhesion after TNF*α* pretreatment is mediated in an overlapping fashion involving CXCR2 and E selectin [30].

**Figure 2:**
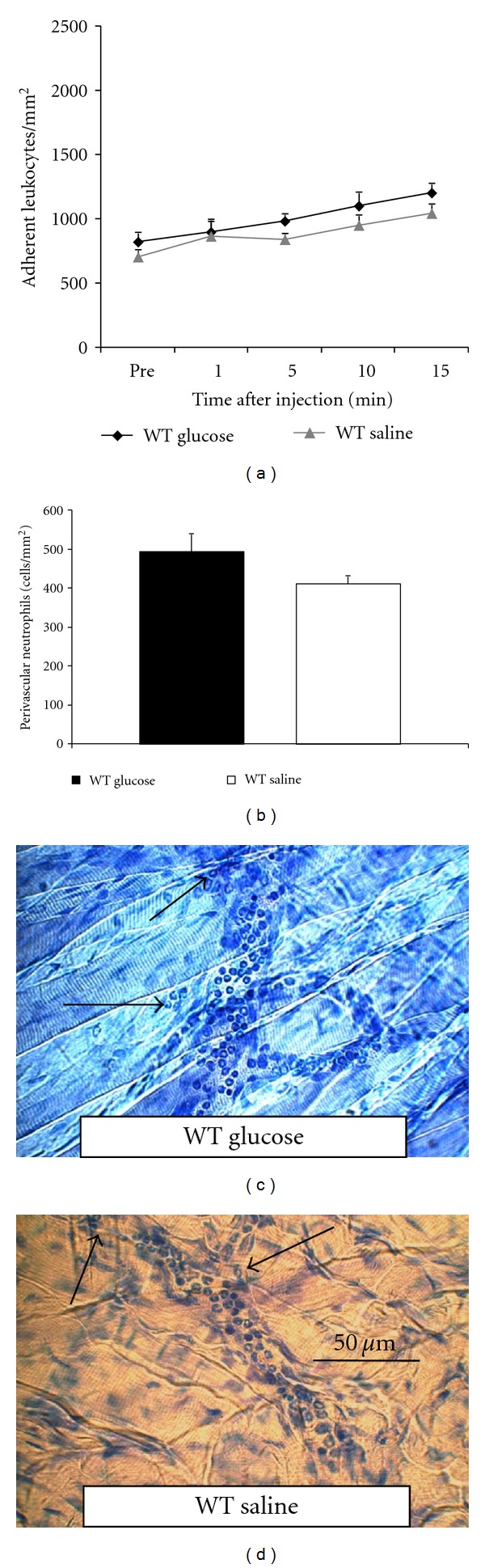
Leukocyte adhesion (number of adherent cells/mm^2^) in mouse cremaster muscle venules and leukocyte transmigration (perivascular neutrophils/mm^2^ surface area) in cremaster muscle whole mounts in response to glucose during TNF*α*-induced inflammation. (a) Leukocyte adhesion was observed in TNF*α*-stimulated cremaster muscle venules of wild-type mice (17 venules in 5 mice) before and during 15 minutes after intravenous injection of 0.5 g/kg glucose and compared to injection of normal saline (9 venules in 4 mice). (b) Giemsa-stained whole mounts of TNF*α*-treated cremaster muscles of WT mice were analyzed for the number of perivascular neutrophils after injection of glucose or normal saline. Representative micrographs are depicted in Figures 2(c) and 2(d). Reference bar is presented in Figure 2(d). Arrows point to extravasated leukocytes. All values are presented as mean ± SEM. Significances are indicated by the asterisks (*P < 0.05 versus WT control mice).

Microvascular parameters for the groups are presented in Table 2 and show similar vessel diameters, centerline velocities, wall shear rates, and WBC counts. Administration of 0,5 g/kg glucose did not significantly increase leukocyte adhesion in the TNF*α* model when compared to injection of normal saline (Figure 2(a)).

To investigate whether glucose alters neutrophil transmigration, we performed Giemsa staining of whole mounts of TNF*α*-treated cremaster muscles and classified leukocytes into neutrophils, eosinophils, and mononuclear cells as described [17]. In mice injected with glucose, the number of extravasated neutrophils was hardly increased compared to normal saline-treated mice, indicating that proinflammatory stimulation with TNF*α* is not further enhanced by glucose (Figures 2(b)–2(d)).

Thus, glucose exerts proinflammatory effects with regard to leukocyte recruitment in the short-term trauma model, but not in the long-term TNF*α* model. 

We hypothesize that in contrast to the short term model of trauma-induced inflammation TNF*α* induces a very strong NF*κ*B-triggered proinflammatory stimulation over 3h that is not further augmentable by glucose administration. This theory is supported by the fact that the level of leukocyte adhesion and transmigration is about the same after TNF*α* stimulation compared to trauma-induced inflammation plus glucose. 

### 3.3. Role of ICAM1 and LFA1 for Glucose-Stimulated Leukocyte Adhesion and Transmigration during Trauma-Induced Inflammation

Using ICAM1-KO and LFA1-KO mice we addressed the question whether ICAM1 and LFA1 play a role for the observed glucose-induced effects during leukocyte recruitment during trauma-induced inflammation (hemodynamic and microvascular parameters are presented in Table 3). In line with previous reports [31, 32] we found comparable baseline leukocyte adhesion in cremaster muscle venules of WT, LFA1-KO, and ICAM1-KO mice. In contrast to WT mice, injection of 0.5 g/kg glucose in ICAM1^−/−^ and in LFA1^−/−^ mice did not lead to significant changes of adherent leukocytes compared to baseline values (Figure 1(a)). Accordingly, transmigration of neutrophils as observed in giemsa-stained cremaster muscle whole mounts of ICAM1-KO and in LFA1-KO mice was similar after administration of glucose compared to normal saline (Figures 1(b), 1(f), and 1(g)). These findings suggest that ICAM1 and LFA1 are crucially involved in mediating glucose-induced leukocyte recruitment, which is in line with previous observations [8, 33].

### 3.4. Role of Chemokine CXCL1-CXCR2 Pathway for Glucose-Stimulated Leukocyte Adhesion and Transmigration during Trauma-Induced Inflammation

In order to gain further insight into underlying mechanisms, we next investigated the role of the CXCL1-CXCR2 pathway for glucose-triggered leukocyte recruitment in our experimental setting. We and others demonstrated that injection of CXCR2-binding chemokine CXCL1 induces a significant increase of leukocyte adhesion which is mostly attributed to LFA1 activation and its subsequent ICAM1 binding [23, 31].

We first asked whether systemically injection of 600 ng CXCL1 in addition to 0.5 g/kg glucose would be able to further enhance leukocyte adhesion and transmigration during trauma-induced inflammation (hemodynamic and microvascular parameters are presented in Table 3). However, the number of adherent and transmigrated cells was not further increased in glucose-injected mice after costimulation with CXCL1 compared to glucose injection only (Figures 1(a), 1(b), and 1(h)), indicating that CXCL1 might be involved in glucose-mediated leukocyte recruitment. Next, we used Pertussis toxin (PTx) to unspecifically block the chemokine receptor CXCR2 by disruption of G-protein-coupled receptor signaling [23]. Notably, PTx pretreatment completely abolished leukocyte adhesion or transmigration in response to glucose in our experimental model (Figures 1(a), 1(b), and 1(e)) suggesting that the CXCL1-CXCR2 pathway is involved in mediating glucose-induced leukocyte recruitment.

### 3.5. Glucose-Dependent Expression of LFA1 and MAC1 on Bone Marrow-Derived Neutrophils

**Figure 3:**
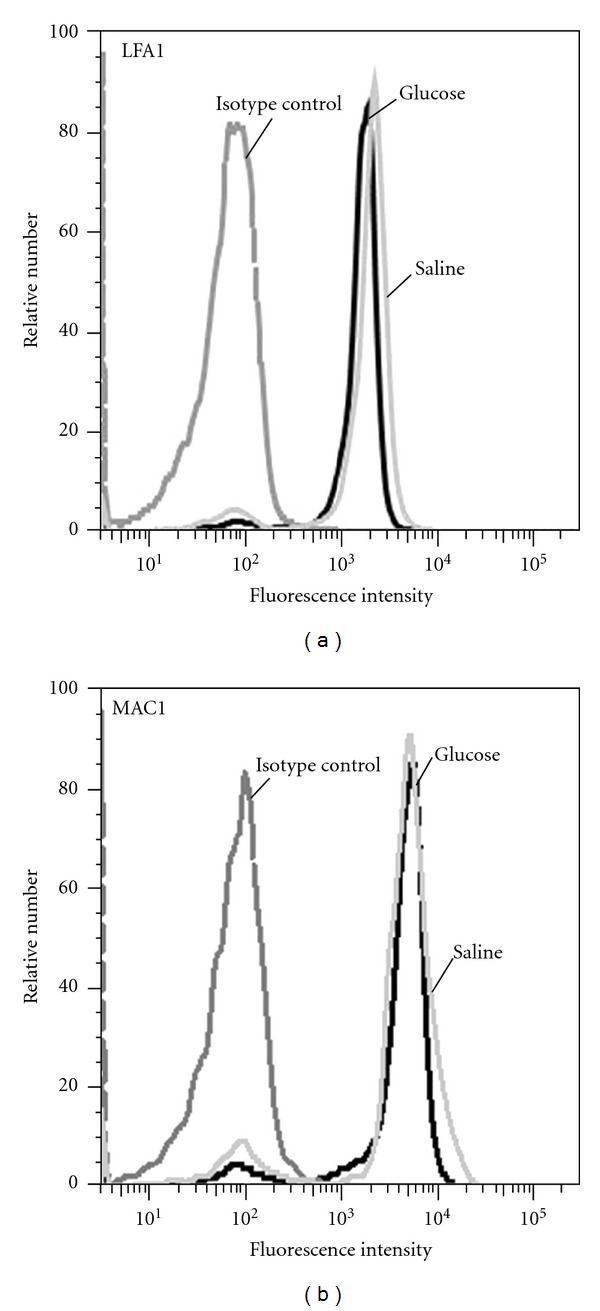
Glucose-dependent expression of LFA1 and MAC1 on neutrophils. Surface expression of LFA1 (a) and MAC1 (b) on bone marrow-derived neutrophils (*n* = 3 mice) after stimulation with glucose (10 mg per 10^6^ leukocytes/mL, 1 h at 37°C) was compared to unstimulated controls (b). Representative histograms are shown from 3 separate experiments.

To further explore underlying mechanisms for the observed glucose-induced effects we analyzed the expression of the *β*_2_ integrins LFA1 and MAC1 on neutrophils in response to glucose using flow cytometry. As depicted in Figure 3, expression of LFA1 and MAC1 on bone marrow-derived mouse neutrophils was not altered after incubation with 10 mg/mL glucose for 15 minutes when compared to control neutrophils. This finding indicates that upregulation of *β*_2_ integrins does not account for the observed glucose-induced augmentation of leukocyte recruitment. Therefore, the changes of leukocyte recruitment are unlikely to be attributable to leukocyte-driven mechanism, as signaling of glucose-incubated leukocytes is identical to leukocytes incubated with saline. Glucose-evoked changes of integrin avidity or activity are difficult to exclude in our flow cytometric approach, as respective antibodies are not available in mice.

### 3.6. Leukocyte Adhesion during Dynamic Microflow Chamber Experiments

**Figure 4:**
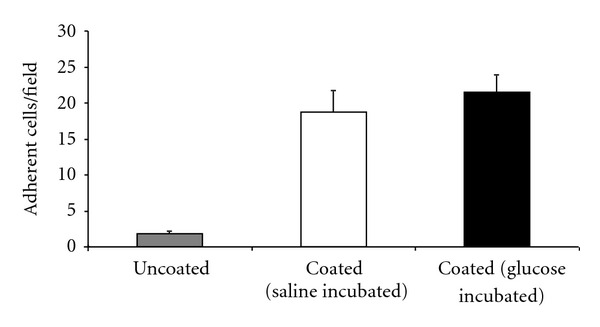
Comparison of adherent cells in microflow chambers perfused with glucose or saline. Microflow chambers were coated with P selectin, ICAM1, and KC and constantly perfused with isolated glucose-stimulated or saline control bone marrow neutrophils of LysEGFP mice (*n* = 4). Adhesion of leukocytes in fully coated flow chambers was compared to uncoated flow chambers.

Next, we aimed to further dissect leukocyte- from endothelium-driven mechanisms mediating glucose-induced adhesion of leukocytes and performed flow chamber experiments. Therefore, microflow chambers were coated with P selectin, ICAM1, and KC and constantly perfused with isolated glucose-stimulated or saline control bone marrow neutrophils of *LysEGFP* mice. Under control conditions we observed a significant number of adherent GFP-positive cells (mainly neutrophils) in fully coated flow chamber compared to uncoated flow chambers (Figure 4). Noteworthy, glucose stimulation (10 mg/mL for 15 minutes) did not affect leukocyte adhesion in this dynamic flow chamber approach, indicating that leukocyte-born mechanisms are unlikely to be connected to glucose-induced changes of leukocyte recruitment.

### 3.7. Glucose-Dependent ICAM1 Expression in Surgically Prepared Cremaster Muscle Venules

We next ask about the impact of glucose on the regulation of endothelial adhesion molecules. Since interaction of LFA1 with ICAM1 is involved in mediation of glucose-induced effects of leukocyte recruitment, we assessed endothelial ICAM1 expression in postcapillary venules of trauma-stimulated cremaster muscles using immunohistochemistry.

**Figure 5:**
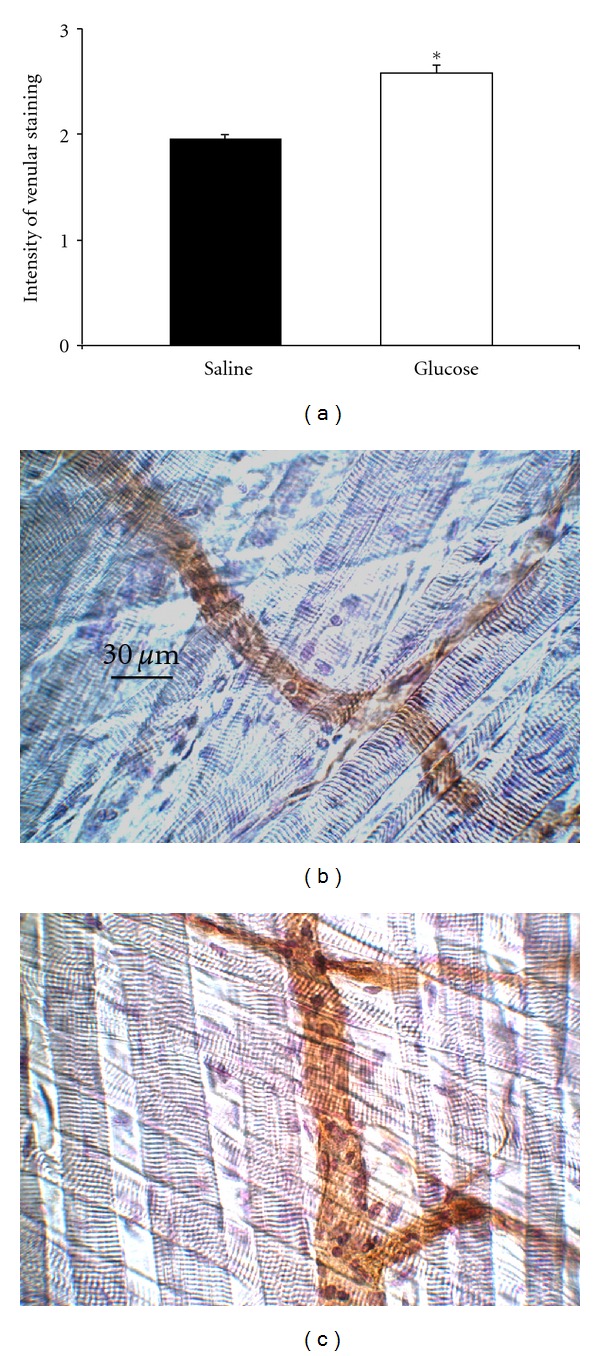
Glucose-induced expression of ICAM1 in surgically prepared cremaster muscle venules. Immunostaining was conducted to assess endothelial expression of ICAM1 in postcapillary venules of cremaster muscles obtained 15 minutes after exteriorization and injection of 0.5 g/kg glucose or the equivalent volume of normal saline. Application of primary antibody was performed i.v. before harvesting the cremaster muscle in order to stain ICAM1 on the endothelial surface. Biotinylated secondary antibody, peroxidase-conjugated streptavidin, and diaminobenzidine (DAB) were used to detect endothelial expression of ICAM1 as brown signal. Counter-staining was performed by Mayer's hemalaun. Intensity of venular anti-ICAM1 immunostaining during trauma-induced inflammation was analyzed semiquantitatively and presented as mean ± SEM (a; 0 = no, 1 = weak, 2 = medium, 3 = strong signal; at least 3 mice/group). In addition, representative images of saline- and glucose-treated mice are depicted in (b) and (c), respectively. Reference bar is shown in (b) and represents 30 *μ*m. Significant differences (*P* < 0.05) are indicated by the asterisk.

Endothelial ICAM1 expression in cremaster muscle venules of glucose treated mice was significantly increased compared to control mice (Figure 5, also illustrated by micrographs). These findings confirm previous observations of Azcutia et al. and others [8, 33], strongly suggesting that glucose-dependent enhancement of leukocyte recruitment is at least in part entailed to ICAM1 upregulation. However, future studies are necessary to investigate whether glucose regulates other endothelial leukocyte adhesion molecules acting in concert with ICAM1. In addition, humoral factors cytokines and chemokines could be involved in the glucose-induced proinflammatory responses, as described by Azcutia et al. and Ling et al. [8, 34].

Based on our presented results we hypothesize that glucose induces CXCL1-CXCR2-dependent activation of LFA1 which in turn binds to upregulated ICAM1 in order to mediate leukocyte recruitment. However, up to date it remains unclear whether and how glucose-dependent signaling, that is, glucose receptors or channels are involved in upstream signalling pathways. 

To our knowledge, this is the first study that investigated the immediate effects of intravenously administered glucose on the leukocyte recruitment cascade *in vivo*, so that our results may stimulate future studies further investigating underlying mechanisms of the observed glucose-dependent proinflammatory signaling.

## 4. Conclusion

Leukocyte adhesion and transmigration are strongly augmented by intravenous glucose injection during trauma-induced inflammation but not during 3h-TNF*α* stimulation. The glucose-induced leukocyte recruitment is mediated by CXCL1-triggered interaction of LFA1 and ICAM1. Thus, our results also indicate that the proinflammatory properties of glucose are stimulus-dependent and might open new perspectives for the development of strategies targeting hyperglycemia-related inflammatory conditions.

## Supplementary Material

Supplemental Figure: Leukocyte adhesion after varying doses of D-glucose (0.25 g–1 g/kg body weight) was compared to injection of normal saline and the osmotic control L-glucose. A dose of 0.5 g/kg D-glucose lead to significantly enhanced leukocyte adhesion, whereas this effect was neither observed after injection of normal saline nor after injection of L-glucose. An augmentation of the glucose dose to 1 g/kg caused a more prominent increase of leukocyte adhesion when compared to 0.5 g/kg, stating that the effects of glucose on the leukocyte adhesion are dose-dependent.
Supplemental Table: Blood glucose concentration was measured at different time points after injection of varying doses of D-glucose (0.25 g–1 g/kg body weight) and compared to the injection of normal saline or the osmotic control L-glucose. A dose of 0.5 g/kg was sufficient to significantly rise blood glucose concentration, an effect that was even more pronounced after injection of 1 g/kg glucose and irreproducable after injection of normal saline or L-glucose. This observation demonstrates that intravenously injected glucose has an impact on systemic blood glucose concentration that is influenced in a dose-dependent manner.

## Figures and Tables

**Table 1:** Hemodynamic and microvascular parameters of cremaster muscle venules before and after stimulation with glucose in the trauma model. Vessel diameter, centerline velocity, and wall shear rate are displayed before and after the intravenous administration of different doses of glucose and compared to the application of the equivalent volume of normal saline and the biologically inactive L-glucose. Experiments are presented as mean ± SEM; n.s.: not significant, stating no differences of hemodynamic parameters among the different doses and during the time course after glucose/saline injection.

	Mice	Venules	Diameter	Centerline velocity	Wall shear rate	Systemic leukocyte counts
	*N*	*n*	(*μ*m)	(*μ*m/s)	(s^−1^)	(/*μ*L)
Saline injection						

Pre-saline	4	18	28 ± 1	2200 ± 100	1900 ± 100	6300 ± 300
5′ after saline	4	18	28 ± 1	2300 ± 100	1900 ± 100	6200 ± 200
15′ after saline	4	18	28 ± 1	2400 ± 100	2000 ± 100	5900 ± 400
			n.s.	n.s.	n.s.	n.s.

Injection of 0,25 g/kg glucose						

Pre-glucose	3	12	30 ± 1	2400 ± 100	2400 ± 100	4300 ± 200
5′ after glucose	3	12	30 ± 1	2400 ± 100	2000 ± 100	4300 ± 200
15′ after glucose	3	12	30 ± 1	2400 ± 100	2000 ± 100	4400 ± 200
			n.s.	n.s.	n.s.	n.s.

Injection of 0,5 g/kg L-glucose						

Pre-glucose	5	26	29 ± 1	2300 ± 100	1900 ± 100	4900 ± 200
5′ after glucose	5	13	29 ± 1	2300 ± 100	1900 ± 100	4800 ± 200
15′ after glucose	5	18	29 ± 1	2300 ± 100	2000 ± 100	4900 ± 200
			n.s.	n.s.	n.s.	n.s.

Injection of 0,5 g/kg glucose						

Pre-glucose	16	64	28 ± 1	2200 ± 100	1900 ± 100	6500 ± 100
5′ after glucose	16	64	28 ± 1	2300 ± 100	2000 ± 200	6200 ± 200
15′ after glucose	16	64	28 ± 1	2400 ± 100	2000 ± 100	6400 ± 100
			n.s.	n.s.	n.s.	n.s.

Injection of 1 g/kg glucose						

Pre-glucose	4	10	27 ± 1	2300 ± 100	2200 ± 100	6300 ± 200
5′ after glucose	4	10	27 ± 1	2400 ± 100	2100 ± 100	6300 ± 200
15′ after glucose	4	10	27 ± 1	2300 ± 100	2100 ± 200	6300 ± 200
			n.s.	n.s.	n.s.	n.s.

**Table 2:** Hemodynamic and microvascular parameters of cremaster muscle venules before and after stimulation with glucose in the TNF*α* model. Vessel diameter, centerline velocity, and wall shear rate are displayed before and after the intravenous administration of 0,5 g/kg glucose and compared to the application of the equivalent volume of normal saline. Experiments are presented as mean ± SEM; n.s.: not significant, stating no differences of hemodynamic parameters during the time course after glucose/saline injection.

	Mice	Venules	Diameter	Centerline velocity	Wall shear rate	Systemic leukocyte counts
	*N*	*n*	(*μ*m)	(*μ*m/s)	(s^−1^)	(/*μ*L)
Injection of saline						

Pre-saline	4	9	28 ± 1	1900 ± 100	1800 ± 200	4200 ± 200
5′ after saline	4	9	28 ± 1	1900 ± 100	1800 ± 200	4300 ± 200
15′ after saline	4	9	28 ± 1	1900 ± 100	1800 ± 100	4300 ± 200
				n.s.	n.s.	n.s.

Injection of glucose						

Pre-glucose	5	17	27 ± 1	1900 ± 100	1800 ± 100	3700 ± 200
5′ after glucose	5	17	27 ± 1	2200 ± 100	1900 ± 100	3700 ± 200
15′ after glucose	5	17	27 ± 1	2000 ± 100	1800 ± 100	3900 ± 200
				n.s.	n.s.	n.s.

**Table 3:** Hemodynamic and microvascular parameters of cremaster muscle venules in ICAM1-KO, LFA1-KO, and PTx-pretreated or KC-coinjected WT mice before and after injection 0,5 g/kg glucose in the trauma model. Vessel diameter, centerline velocity, and wall shear rate are displayed before and after the intravenous administration of glucose. Experiments are presented as mean ± SEM; n.s.: not significant stating no differences of hemodynamic parameters among the different groups and during the time course after glucose/saline injection.

	Mice	Venules	Diameter	Centerline velocity	Wall shear rate	Systemic leukocyte counts
	*N*	*n*	(*μ*m)	(*μ*m/s)	(s^−1^)	(/*μ*L)
WT mice						

Pre-glucose	16	64	28 ± 1	2200 ± 100	1900 ± 100	6500 ± 100
5′ after glucose	16	64	28 ± 1	2300 ± 100	2000 ± 200	6200 ± 200
15′ after glucose	16	64	28 ± 1	2400 ± 100	2000 ± 100	6400 ± 100
			n.s.	n.s.	n.s.	n.s.

Coinjection with KC in WT mice						

Pre-glucose	4	16	27 ± 1	2200 ± 100	2200 ± 100	5900 ± 300
5′ after glucose	4	12	27 ± 1	2300 ± 100	2200 ± 100	6000 ± 200
15′ after glucose	4	14	27 ± 1	2200 ± 100	2000 ± 100	6000 ± 200
			n.s.	n.s.	n.s.	n.s.

PTx-pretreatment in WT mice						

Pre-glucose	4	16	29 ± 1	2200 ± 100	2000 ± 100	5700 ± 300
5′ after glucose	4	15	29 ± 1	2300 ± 200	2000 ± 200	5200 ± 500
15′ after glucose	4	16	29 ± 1	2400 ± 100	2000 ± 100	5300 ± 300
			n.s.	n.s.	n.s.	n.s.

LFA1-KO mice						

Pre-glucose	4	25	28 ± 1	2500 ± 100	2200 ± 100	7600 ± 500
5′ after glucose	4	19	28 ± 1	2400 ± 100	2200 ± 100	8000 ± 300
15′ after glucose	4	20	28 ± 1	2500 ± 100	2200 ± 100	8100 ± 300
			n.s.	n.s.	n.s.	n.s.

ICAM1^−/−^ mice						

Pre-glucose	4	22	27 ± 1	2300 ± 100	2300 ± 100	6000 ± 400
5′ after glucose	4	18	27 ± 1	2500 ± 100	1800 ± 100	6200 ± 500
15′ after glucose	4	21	27± 1	2400 ± 100	2300 ± 100	6100 ± 400
			n.s.	n.s.	n.s.	n.s.
