# Supplementary material for: CXCL1-Triggered Interaction of LFA1 and ICAM1 Control Glucose-Induced Leukocyte Recruitment during Inflammation In Vivo
Source: Mediators Inflamm. 2012 Oct 9;2012:739176. doi: 10.1155/2012/739176 (PMC3474340; doi:10.1155/2012/739176)
Supplement: Supplementary file 1 — Supplemental Figure: Leukocyte adhesion after varying doses of D-glucose (0.25 g–1 g/kg body weight) was compared to injection of normal saline and the osmotic control L-glucose. A dose of 0.5 g/kg D-glucose lead to significantly enhanced leukocyte adhesion, whereas this effect was neither observed after injection of normal saline nor after injection of L-glucose. An augmentation of the glucose dose to 1 g/kg caused a more prominent increase of leukocyte adhesion when compared to 0.5 g/kg, stating that the effects of glucose on the leukocyte adhesion are dose-dependent. Supplemental Table: Blood glucose concentration was measured at different time points after injection of varying doses of D-glucose (0.25 g–1 g/kg body weight) and compared to the injection of normal saline or the osmotic control L-glucose. A dose of 0.5 g/kg was sufficient to significantly rise blood glucose concentration, an effect that was even more pronounced after injection of 1 g/kg glucose and irreproducable after injection of normal saline or L-glucose. This observation demonstrates that intravenously injected glucose has an impact on systemic blood glucose concentration that is influenced in a dose-dependent manner. [file 739176.f1.pdf]

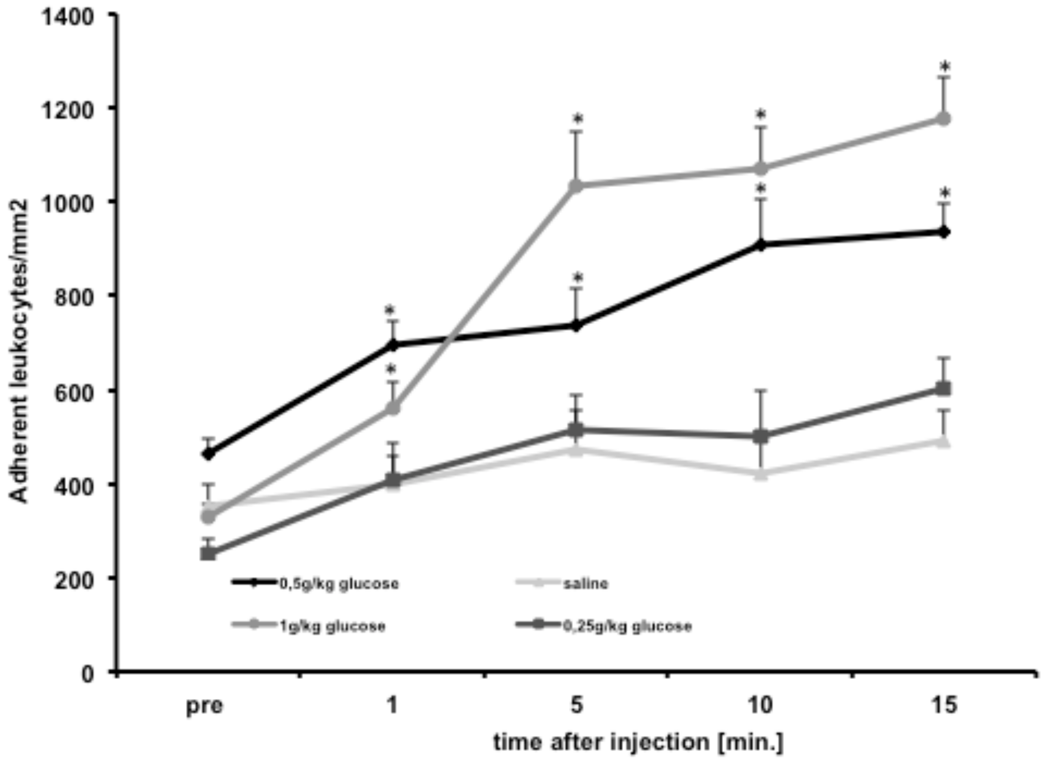

Supplemental Figure 1: Leukocyte adhesion (number of adherent cells/mm^2) in mouse cremaster muscle venules after varying doses of glucose or normal saline injection in trauma-induced inflammation. Leukocyte adhesion was observed in trauma-stimulated cremaster muscle venules of wild type mice injected with 0,25g/kg glucose (12 venules in 3 mice), 0,5g/kg glucose (64 venules in 16 mice) and 1g/kg glucose (10 venules in 4 mice). The number of adherent leukocytes was quantified before and during 15 minutes after intravenous injection of glucose and compared to injection of normal saline (18 venules in 4 mice). Significant differences ($p < 0.05$) are indicated by the asterisks

Supplemental Table 1. Course of blood glucose concentration. Blood glucose concentration was measured in blood samples obtained via carotid artery before and at different time points after intravenous injection of variable doses of glucose or saline as control. All parameters are presented as mean ± SEM; **#** displays a significant difference when compared to initial blood glucose concentration.

		Blood glucose concentration (mg/dl)	
Dose of glucose (g/kg)	*Before injection*	*1' after injection*	*10' after injection*
0	270 ± 30	290 ± 20	320 ± 20
0,25	210 ± 50	270 ± 50	280 ± 70
0,5	250 ± 20	430 ± 20 (**#**)	320 ± 10
1,0	280 ± 30	500 ± 100 (**#**)	490 ± 90 (**#**)
